# Smartphone fluorescence imager for quantitative dosimetry of protoporphyrin-IX-based photodynamic therapy in skin

**DOI:** 10.1117/1.JBO.25.6.063802

**Published:** 2019-12-09

**Authors:** Alberto J. Ruiz, Ethan Phillip M. LaRochelle, Jason R. Gunn, Sally M. Hull, Tayyaba Hasan, M. Shane Chapman, Brian W. Pogue

**Affiliations:** a Dartmouth College, Thayer School of Engineering, Hanover, New Hampshire, United States; b Massachusetts General Hospital, Harvard Medical School, Wellman Center for Photomedicine, Boston, Massachusetts, United States; c Geisel School of Medicine, Department of Surgery, Hanover, New Hampshire, United States

**Keywords:** fluorescence imaging, smartphone, photodynamic therapy, dosimetry, actinic keratosis, treatment planning

## Abstract

**Significance:** While clinical treatment of actinic keratosis by photodynamic therapy (PDT) is widely practiced, there is a well-known variability in response, primarily caused by heterogeneous accumulation of the photosensitizer protoporphyrin IX (PpIX) between patients and between lesions, but measurement of this is rarely done.

**Aim:** Develop a smartphone-based fluorescence imager for simple quantitative photography of the lesions and their PpIX levels that can be used in a new clinical workflow to guide the reliability of aminolevulinic acid (ALA) application for improved lesion clearance.

**Approach:** The smartphone fluorescence imager uses an iPhone and a custom iOS application for image acquisition, a 3D-printed base for measurement standardization, an emission filter for PpIX fluorescence isolation, and a 405-nm LED ring for optical excitation. System performance was tested to ensure measurement reproducibility and the ability to capture photosensitizer accumulation and photobleaching in pre-clinical and clinical settings.

**Results:** PpIX fluorescence signal from tissue-simulating phantoms showed linear sensitivity in the 0.01 to 2.0 *μ*M range. Murine studies with Ameluz^®^ aminolevulinic acid (ALA) gel and initial human testing with Levulan^®^ ALA cream verified that *in-vivo* imaging was feasible, including that PpIX production over 1 h is easily captured and that photobleaching from the light treatment could be quantified.

**Conclusions:** The presented device is the first quantitative wide-field fluorescence imaging system for PDT dosimetry designed for clinical skin use and for maximal ease of translation into clinical workflow. The results lay the foundation for using the system in clinical studies to establish treatment thresholds for the individualization of PDT treatment.

## 1 Introduction

Clinical use of protoporphyrin-IX (PpIX)-based photodynamic therapy (PDT) is widespread for treatment of actinic keratosis (AKs) and increasingly adopted for treatment of nonmelanoma skin cancers, due to its effectiveness, safety, and cosmetic results.^1^ PpIX-based skin PDT involves the topical application of the prodrug aminolevulinic acid (ALA) or methyl aminolevulinate (MAL) to selectively generate the photosensitizer PpIX in the lesion; after incubation, the lesion is commonly irradiated with light where localized cytotoxicity is induced via reactive molecular species.^2^ While there is widespread use of skin PDT, the complete clearance rate of AKs can vary from ∼59% to 91%,^3^ where the origins of this variability in response is thought to come from the heterogeneity of drug absorption and PpIX accumulation between patients and between lesions.^4^^,^^5^ Dosimetry of PpIX at the point of care can help guide critical decisions in PDT, given that initial accumulation and PDT-induced photobleaching serve as strong indicators for patient outcomes in treatment.^6^^–^^8^ Nonetheless, in the clinical setting, dosimetry is rarely performed during PDT treatments to personalize incubation times and light doses, primarily due to a lack of practical dosimetry devices and limited predictive methods for corrective action if the PpIX levels look low. The sparse use of dosimetry in clinical PDT studies has led to a restricted understanding of optimal treatment parameters that are further confounded by the adoption of newer treatment protocols, such as daylight PDT with minimization of the ALA incubation time. Nonetheless, if it was known that there were suboptimal levels of PpIX, there is a range of things that could be done to mitigate this, including increased incubation times, increased skin temperature,^9^ reapplication of ALA after curettage,^10^ differentiation therapy,^11^ and fractionated light treatment.^12^ Wide-field imaging of PpIX shows promise in guiding PDT by providing easily accessible drug production information at the point of care for these treatment enhancements, which may reduce repeat PDT visits or the added cost of future surgical treatments.^4^^,^^13^

Point-probe and wide-field PpIX fluorescence have been used in a limited number of clinical studies for visual localization of lesions and to correlate photosensitizer accumulation and photobleaching to treatment outcomes.^5^^–^^8^^,^^14^^–^^32^ Devices that have achieved translational clinical use, for example FluoDerm (Dia Medico ApS, Denmark), are used primarily for qualitative visualization and are unable to provide quantitative wide-field dosimetry of a lesion, which is necessary to account for the millimeter-level variations in photosensitizer accumulation. Devices capable of quantitative dosimetry have not achieved translational use mainly due to problems with measurement reproducibility, system cost, and ease of use. Point-probe measurements suffer from reproducibility issues due to variability of PpIX accumulation along the surface of the skin and sensitivity to applied probe pressure during measurement. Wide-field devices suffer from measurement inconsistencies related to system warm-up, measurement angle, and varying lighting environments.^32^

In this work, we present the first quantitative wide-field fluorescence imaging system for PpIX-PDT dosimetry designed for clinical treatment of the skin and for maximal ease in translation into clinical workflow. The handheld system consists of a custom application for image acquisition and analysis based on an iPhone 6s platform, with physical light management from a 3D-printed base that includes a long-pass emission filter for signal isolation, and a custom 405-nm wavelength light source and electronics for PpIX excitation. In the following sections, an overview is provided of the dosimeter design, followed by system characterization and preclinical and clinical results.

## 2 Materials and Methods

### 2.1 Smartphone Dosimeter

**Fig. 1:**
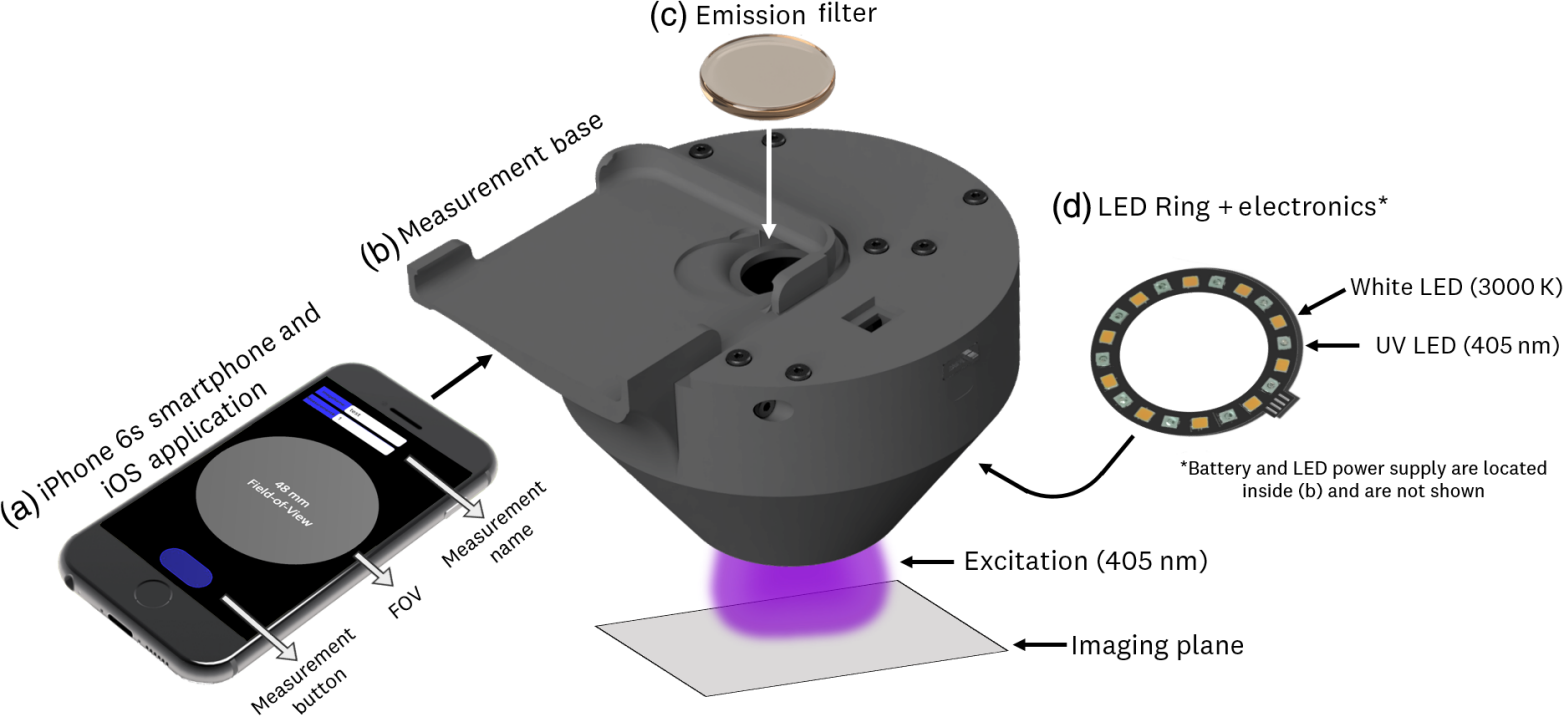
Schematic representation of the dosimeter system: (a) iPhone 6s smartphone and custom iOS application for RAW image acquisition and image analysis; (b) 3D-printed measurement base for system integration and measurement distance and light leakage standarization; (c) 600-nm wavelength long-pass emission filter for PpIX signal isolation; and (d) 405-nm wavelenth LED ring and electronics for excitation and to provide rechargeability in a modular package. FOV, field of view.

The fluorescence imaging system consists of an iPhone 6s smartphone and an application for streamlined image acquisition, a 3D-printed base for measurement standardization and system integration, an emission filter for PpIX signal isolation, and a custom LED ring and electronics for PpIX excitation. A schematic of the system is presented in Fig. 1. The following sections briefly summarize the design and function of each component; a detailed description of the system design, beyond the scope of this paper, can be found in Ruiz et al.^33^

#### 2.1.1 Smartphone and iOS application

The 12MP back-facing camera of the iPhone 6s is used for imaging, given its ability to manually control camera parameters and provide the RAW pixel intensity values in 12-bit format. A native iOS application was developed for providing fixed imaging parameters, generating RAW output images, and developing a streamlined clinical workflow. Current implementation of the application fixes the imaging parameters via software and only allows, through the visual interface, for the user to name the image and initiate a measurement through a single button press. The result of this measurement is both RAW and JPEG images taken at the fixed, software-determined, imaging parameters.

#### 2.1.2 3D-printed base

The 3D-printed base is used for system integration and measurement standardization by physically blocking room light with a black plastic printing material. Furthermore, a cushioned ring is attached to the exit aperture, which allows coupling to the skin. The measurement base fixes the detector-to-imaging plane distance for consistent quantitative images. The base consists of three components: the measurement cone, electronics bay, and top cover. These components are fastened together using M3 screws. It is worth emphasizing that the design of the 3D-printed base, alongside the cushioned ring, forms an isolated lighting environment that allows the system to overcome measurement repeatability issues faced by previously reported systems, including variation in measurement angles and lighting environments.

The detector-to-imaging plane distance was set at 72 mm, which is approximately the minimum focusing distance achievable by the iPhone 6s back-facing camera. The output aperture of the measurement base provided a circular field of view of 48 mm.

#### 2.1.3 Emission filter

The emission filter was chosen to block the 405-nm excitation light and to isolate the PpIX fluorescent signal (600 to 700 nm). For filter selection, dielectric, color-glass, and gel filters were tested. The decision criteria of a filter were based on maximizing emission throughput, while minimizing filter autofluorescence. Both dielectric and gel filters were assessed for the final system design. The current design used a 600-nm long-pass dielectric filter (600LP RapidEdge, Omega Optical Inc., Brattleboro, Vermont), which, based on *in-vivo* testing, led to a maximal PpIX signal-to-skin autofluorescence signal ratio.

#### 2.1.4 LED ring and power electronics

The system uses a custom LED ring to provide a phone-independent uniform light source for UV excitation and white-light illumination. The ring consists of 405-nm LEDs (Vishay VLMU3100) and 3000 K white LEDs (Cree J-Series JE2834AWT) providing light intensities of 4.5 and $2.6\;{\mathrm{mW}/\mathrm{cm}}^{2}$ at the measurement output, respectively. The LEDs are mounted on a 1.6-mm aluminum metal core printed circuit board (2-oz Cu trace) to provide passive thermal management.

The system power electronics consist of a 3.7-V lithium polymer battery (#259, Adafruit Inc., New York City), a USB battery manager (#2011, Adafruit Inc., New York City), and a constant current driver (MIC2287, Microchip Technology Inc., Chandler, Arizona) to provide power to the LED ring (40 mA, constant current) and enable stable light output and rechargeability in a modular package. Current electronics implementation utilizes a physical switch mounted on the side of the 3D-printed base for manual operation of the light source.

### 2.2 Dosimeter Calibration and Characterization

To ensure measurement reproducibility, the dosimeter’s spectral, electrical, and pixel linearity characteristics were evaluated. To measure the spectral output of the 405-nm and white LEDs, an Apogee SS-110 VIS spectrometer was used. To measure the emission filter transmission spectra, a Cary 50 Bio UV-VIS spectrophotometer was used.

Pixel linearity of the iPhone camera was tested by varying the driving current of the white LED array to generate increasing irradiances, which were measured by a photometer (S120VC, Thorlabs Inc., Newton, New Jersey). These irradiances were then imaged by the phone on a highly reflective silicone ${\mathrm{TiO}}_{2}$ phantom ($\mu_{a}=0.0078$, $\mu_{s}^{\prime }=1.967$ at 526 nm). The RAW- and JPEG-generated images were analyzed using MATLAB to generate average pixel values versus irradiance curves.

To ensure electrical and emission output stability of the design, which is necessary for reproducible illumination environments, an optical power meter (S120C, Thorlabs, Newton, New Jersey) was used to observe the potential on/off current spikes as well as to test the long-term output stability of the device.

The fluorescence sensitivity of the dosimeter was studied using 1% intralipid phantoms with varying PpIX concentrations (0.01 to 2  μM), alongside a control sample with no PpIX. Intralipid was used as a scattering agent in the mixture. The liquid phantoms were manufactured by combining PpIX powder (P8293-1G, Sigma-Aldrich Inc.) with dimethyl sulfoxide and serially diluting with a 1% intralipid solution manufactured from 20% intralipid stock and 1× phosphate-buffered saline. The serial dilutions were deposited in 5-ml DELRIN wells and imaged with the dosimeter. The PpIX concentrations imaged were 2, 1, 0.75, 0.5, 0.25, 0.1, 0.05, 0.01, and 0  μM. The imaging parameters used for these measurements were RAW images with ISO 400 and exposure set to 1/10th s. Images were preprocessed using the “Adobe DNG Converter” software and analyzed using MATLAB and ImageJ to extract the RAW pixel values.

### 2.3 Preclinical *In-Vivo* Murine Measurements

All animal procedures were carried out following a protocol approved by the Dartmouth College Institutional Animal Care and Use Committee. The nude mouse model was used to examine the dosimeter’s ability to capture *in-vivo* production of PpIX. The measurements were performed on five athymic nude mice fed a low-chlorophyll diet to minimize tissue autofluorescence. For imaging, the mice were placed under anesthesia and onto a heated pad to regulate their body temperature. The temperature was monitored using an IR camera (TG165, FLIR Systems Inc.) and kept in the 32°C to 36°C range. Ameluz^®^ (Biofontera Inc., Boston, Massachusetts), a 10% ALA hydrochloride gel, was topically applied to the backs of the mice so as to contain an ALA-applied area and a control area within the field of view of the imaging system. The mice were kept in a low-light environment to prevent photobleaching of the accumulated PpIX. Images of the mice were taken at 10-min intervals for the first hour with subsequent images at 2 and 3 h of incubation. The mice were kept under anesthesia during the first hour, placed back in the cage, and briefly placed back under anesthesia for imaging at 2 and 3 h of incubation. The imaging parameters used for these measurements were iOS-generated images with ISO 400 and exposure set to 1/10th s. The images were processed in ImageJ, where the red pixels were isolated for region-of-interest (ROI) pixel intensity calculation.

### 2.4 Human Skin Test Measurement

**Fig. 2:**
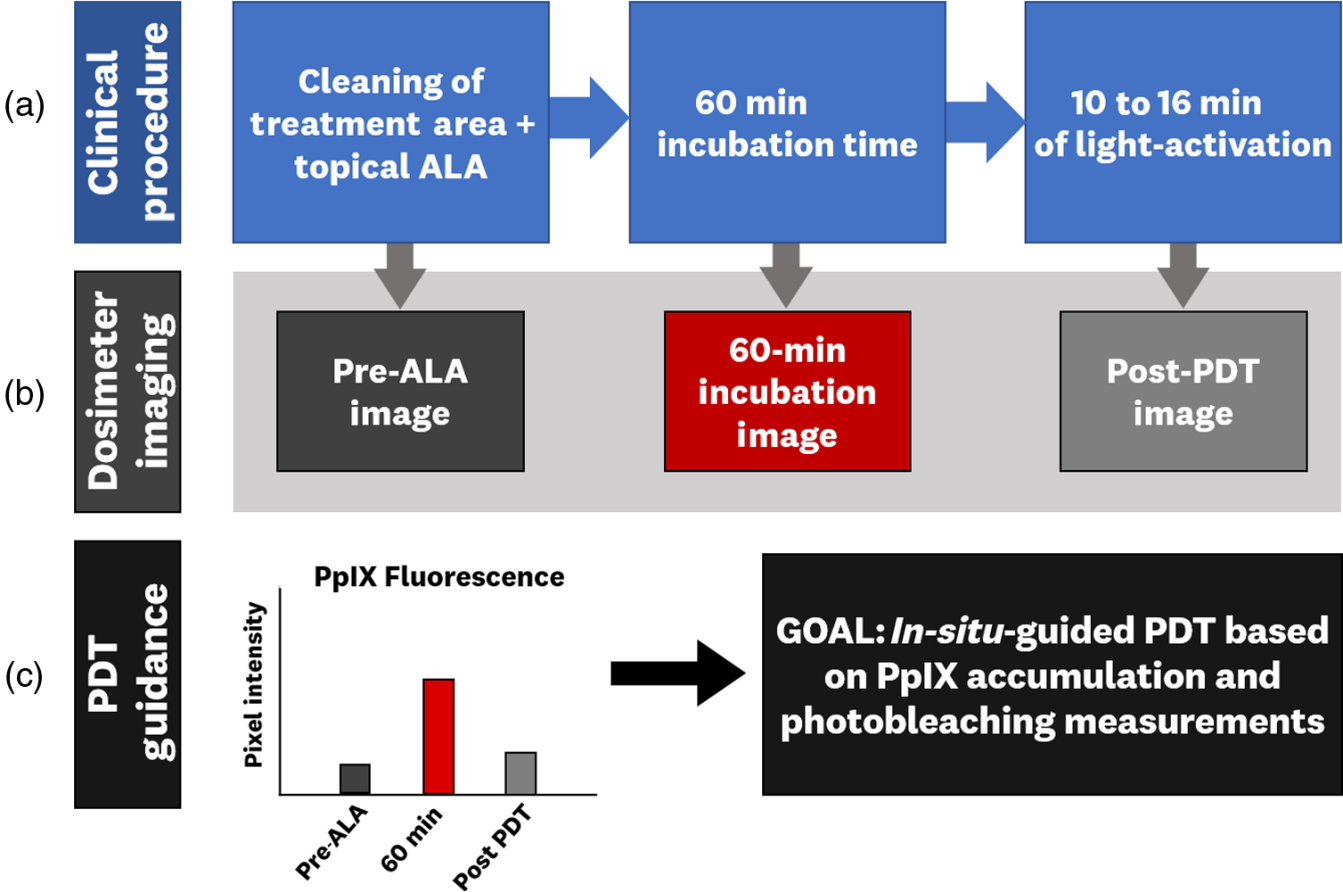
Schematic representation of the clinical workflow and dosimeter imaging integration: (a) clinical procedure used for Levulan in AK-PDT treatment at Dartmouth-Hitchcock Dermatology, (b) dosimeter image acquisition, and (c) proposed PDT guidance from PpIX accumulation and PDT-induced photobleaching measurements.

All human imaging was done on patients who gave informed consent to participate in the study, following the procedures approved in a protocol, approved by the Dartmouth College Institutional Review Board. In this human testing, the system performance was evaluated on three patients undergoing their standard clinical PDT treatment at the Dartmouth-Hitchcock Dermatology Department for treatment of AKs using Levulan^®^ (Sun Pharma Industries Ltd., Mumbai, India), a 20% ALA solution [Fig. 2(a)]. The standard procedure involves treatment of a site (i.e., face, scalp, arms, or legs), which is cleaned using ethanol, and followed by nonoccluded topical application of the ALA. The patient is then transferred to a low-light rest area for a 60-min incubation period. After the 1-h incubation, the patient is given 10 to 16 min of blue-light (Sun Pharma Industries Ltd., Mumbai, India) PDT based on the dermatologist’s prescription. Fluorescence images from a treatment site are gathered before the ALA is applied, at the 60-min incubation, and post-PDT treatment [Fig. 2(b)]. Discomfort levels experienced by the imaged patients were recorded during light irradiation via verbal rating of the pain. The goal of performing dosimeter imaging during treatment is to correlate the measured PpIX accumulation and PDT-induced photobleaching to patient outcomes and generate thresholds that can be used for individualized treatment guidance [Fig. 2(c)]. The dosimetry procedure can be inserted into a regular clinical workflow adding less than a minute to the total treatment time. The imaging parameters used for these measurements were RAW images with ISO 400 and exposure set to 1/10th s. Images were preprocessed using the Adobe DNG Converter software and analyzed using MATLAB and ImageJ to extract the RAW pixel values.

## 3 Results

### 3.1 Dosimeter Calibration and Characterization

#### 3.1.1 Characterization and measurement reproducibility

**Fig. 3:**
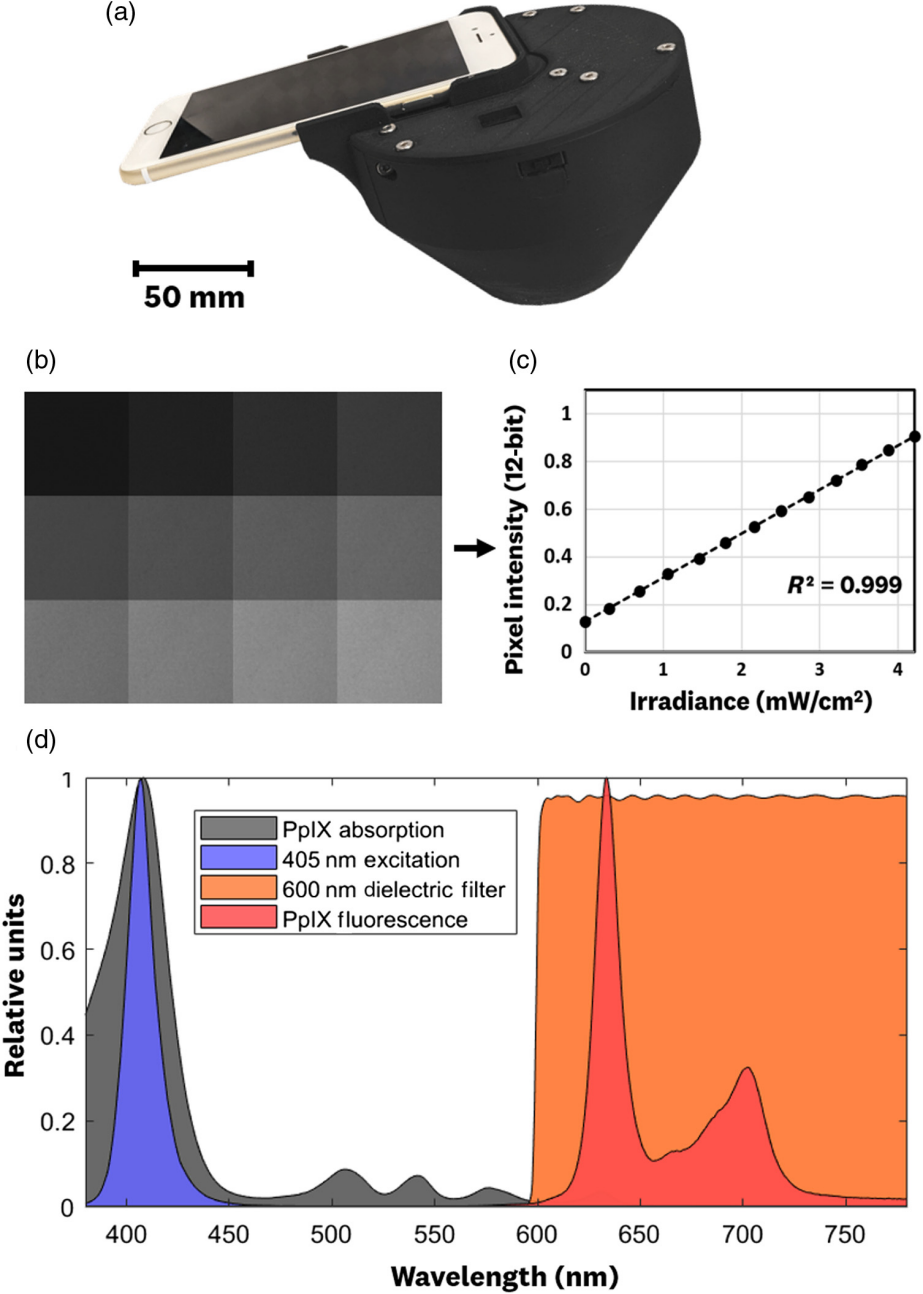
(a) Assembled dosimeter. The system was tested for pixel linearity where (b) varying irradiances at the imaging plane are acquired using the RAW image format and (c) pixel intensity versus irradiance values are plotted; least-squares fit to the data verified a linear relationship between RAW pixel values and image plane irradiance with $R^{2}=0.999$. (d) Spectral plots of PpIX absorption and emission^34^ alongside the system’s 405-nm excitation output and long-pass filter transmission.

The assembled dosimeter [Fig. 3(a)] was tested for pixel linearity and emission output stability. The pixel linearity results [Figs. 3(b) and 3(c)] showed the camera’s ability to recover a linear relationship between pixel intensity and irradiance at the imaging plane when using the RAW image format. The “phone-processed” RGB images were not linear with external irradiance values. Another advantage of using the RAW image format on the iPhone is its 12-bit dynamic range output, compared to the standard 8-bit range for the phone-processed images.

Spectral measurements of the system [Fig. 3(d)] showed great overlap of the 405-nm emission with the PpIX absorption spectra,^34^ blockage of the excitation emission by the spectral filter (OD 4 to 5), and transmission of 95%+ for the PpIX fluorescence signal. It is worth noting that pixel intensity measurements with varying long-pass filters showed a significant transmission of blue-green wavelengths into the red-specific pixels. This meant that, to truly isolate the PpIX signal and prevent excitation wavelengths from “bleeding” into the red-channel signal, the system required a long-pass filter specific for the red wavelengths.

Electrical and emission stability measurements showed an output power stability within 1% for the 405-nm source driven at 40 mA for a 2-min period. Furthermore, on/off cycling of the light source showed flat top-hat profiles with no significant spikes in emission or electrical output.

The irradiance imaging, spectral, electrical, and excitation emission measurements showed the ability of the system to reliably reproduce measurement conditions. The system’s light output stability, imaging linearity, consistent illumination environment, and fixed detector-to-imaging plane distance allows our smartphone dosimeter system to overcome the reproducibility challenges faced by previously reported wide-field fluorescence systems.^32^

#### 3.1.2 Protoporphyrin-IX concentration calibration

**Fig. 4:**
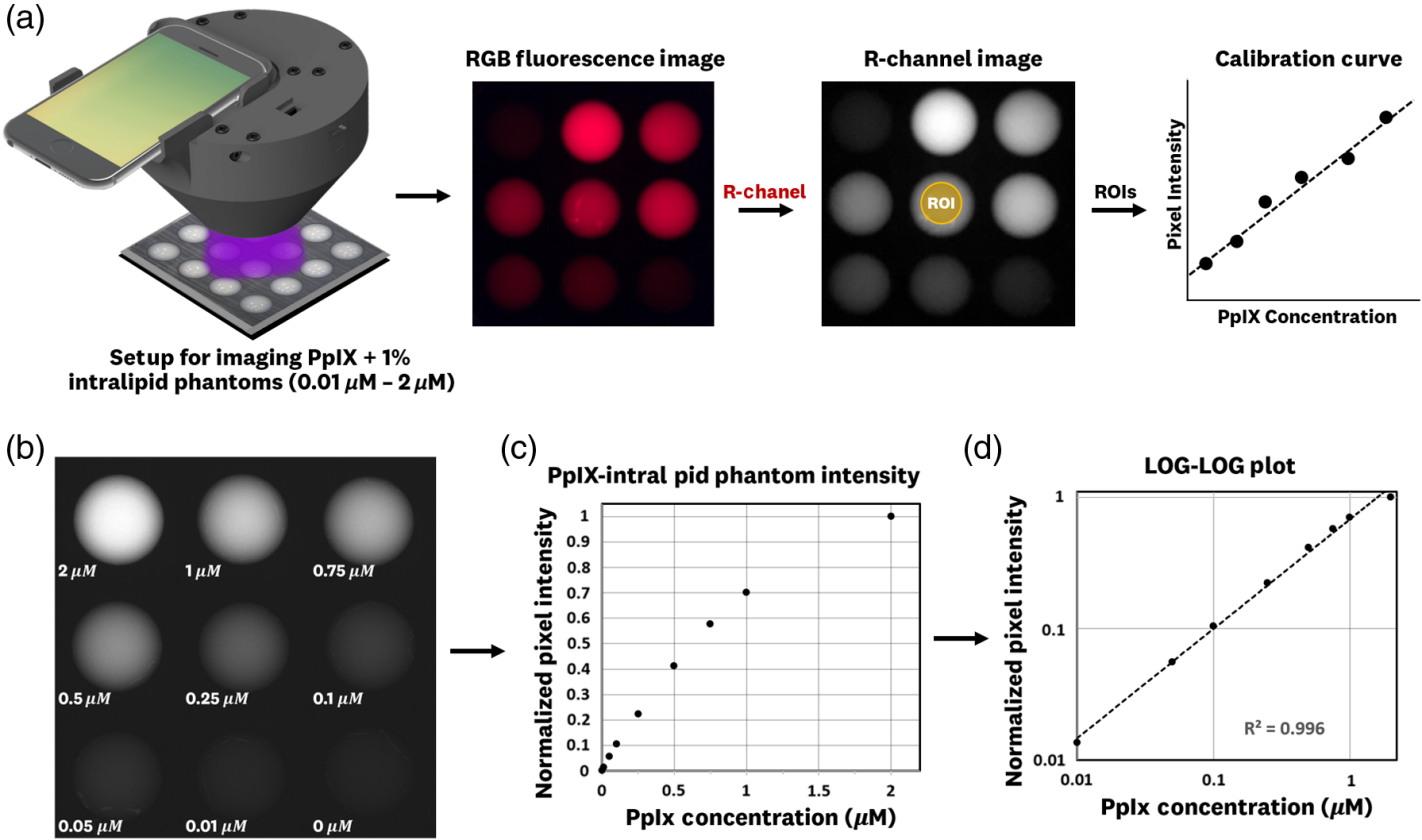
PpIX concentration calibration. (a) Workflow for generating a calibration curve when imaging PpIX+1% intralipid phantoms with the dosimeter system for varying PpIX concentrations in the 0.01 to 2  μM range. (b) Resulting red-pixel intensity images using RAW format; (c) plot of normalized pixel intensity versus Concentration; and (d) log-log plot of data showing high linearity (slope=0.83, $R^{2}=0.996$, least-squares fit) for our measurement range. These results show the system’s ability to measure clinically relevant concentrations with high linearity in pixel response.

Measurement of the dosimeter’s sensitivity involved imaging of 1% intralipid phantoms with varying PpIX concentrations within the 0.01 to 2  μM range, alongside a control, followed by isolating of the red-pixel RAW values to generate a calibration curve. A visual overview of this procedure is shown in Fig. 4(a). The imaging results [Fig 4(b)] showed the dosimeter’s ability to detect the full 0.01 to 2  μM range [Fig. 4(c)], where plotting the data in a log-log scale shows linearity with an $R^{2}=0.996$ for a least-squares fit [Fig. 4(d)].

These results show the dosimeter’s ability to detect clinically relevant PpIX concentrations within the 0.01 to 2  μM range, alongside with high linearity in these measurements (slope=0.83, $R^{2}=0.996$, least-squares fit). The high linearity obtained should enable the creation of skin–PpIX concentration curve calibrations based on the more intricate phantom models and *in-vivo* measurements. These results show the system’s viability to perform quantitative dosimetry and were the basis for proceeding to preclinical and clinical measurements of fluorescence in the next sections.

### 3.2 Preclinical *In-Vivo* Measurements

**Fig. 5:**
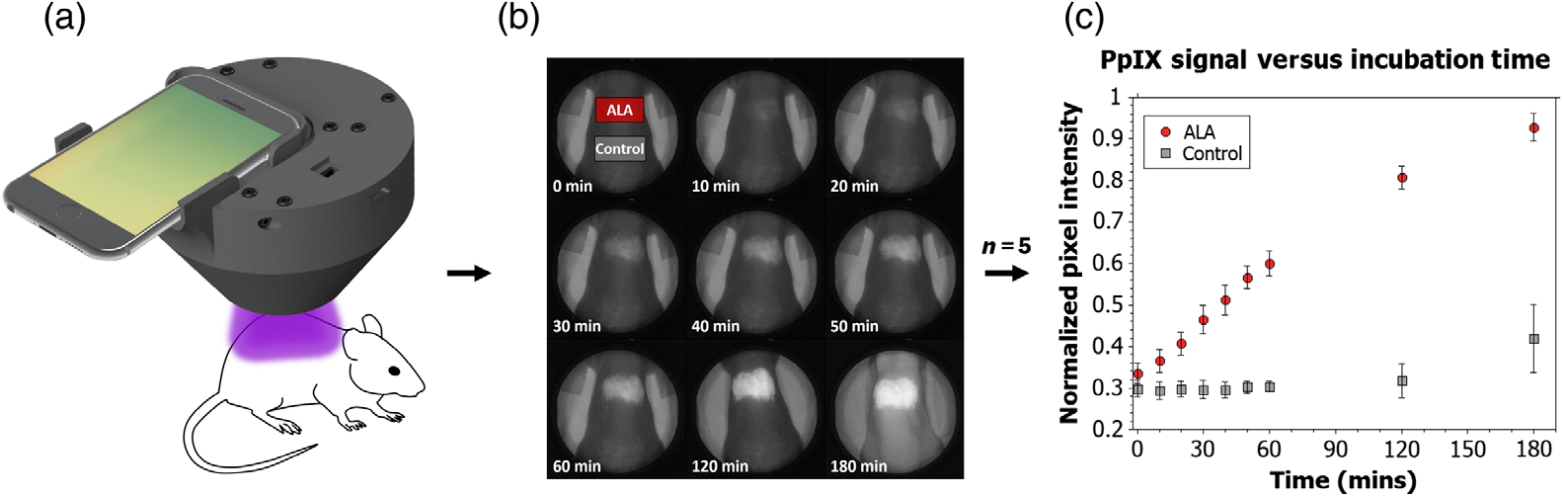
(a) Testing setup for *in-vivo* imaging of PpIX accumulation in athymic nude mice (note: a 3D-printed mount (not shown) was used to standarize the distance to the mice and fix the angle of the dosimeter during measurement). (b) Resulting images that include topically applied ALA and control ROIs. (c) Pixel intensity versus time plot for the ALA and control ROI (averaged for five mice) shows the monotonic increase in fluorescence due to the PpIX accumulation in the ALA region and a relatively flat response in the control region; a moderate increase in fluorescence is observed in the control region at 2 and 3 h of incubation as the mice begin systematic accumulation of PpIX.

Five mice were imaged for tracking the *in-vivo* accumulation of PpIX via topical application of ALA, showing monotonic increase of the fluorescence over the 3 h of incubation (Fig. 5).

In this murine model, it was possible to quantitatively observe the PpIX accumulation in the ALA-applied area as early as 10 min [Fig. 5(c)], with a clear visual accumulation in the 20- to 30-min time frame [Fig. 5(b)]. In contrast, the control region was relatively flat for the first hour with a moderate increase in fluorescence observed at the 2- and 3-hour measurements as the mice begin systematic accumulation of the PpIX [Figs. 5(b) and 5(c)].

### 3.3 Human Clinical Measurement

**Fig. 6:**
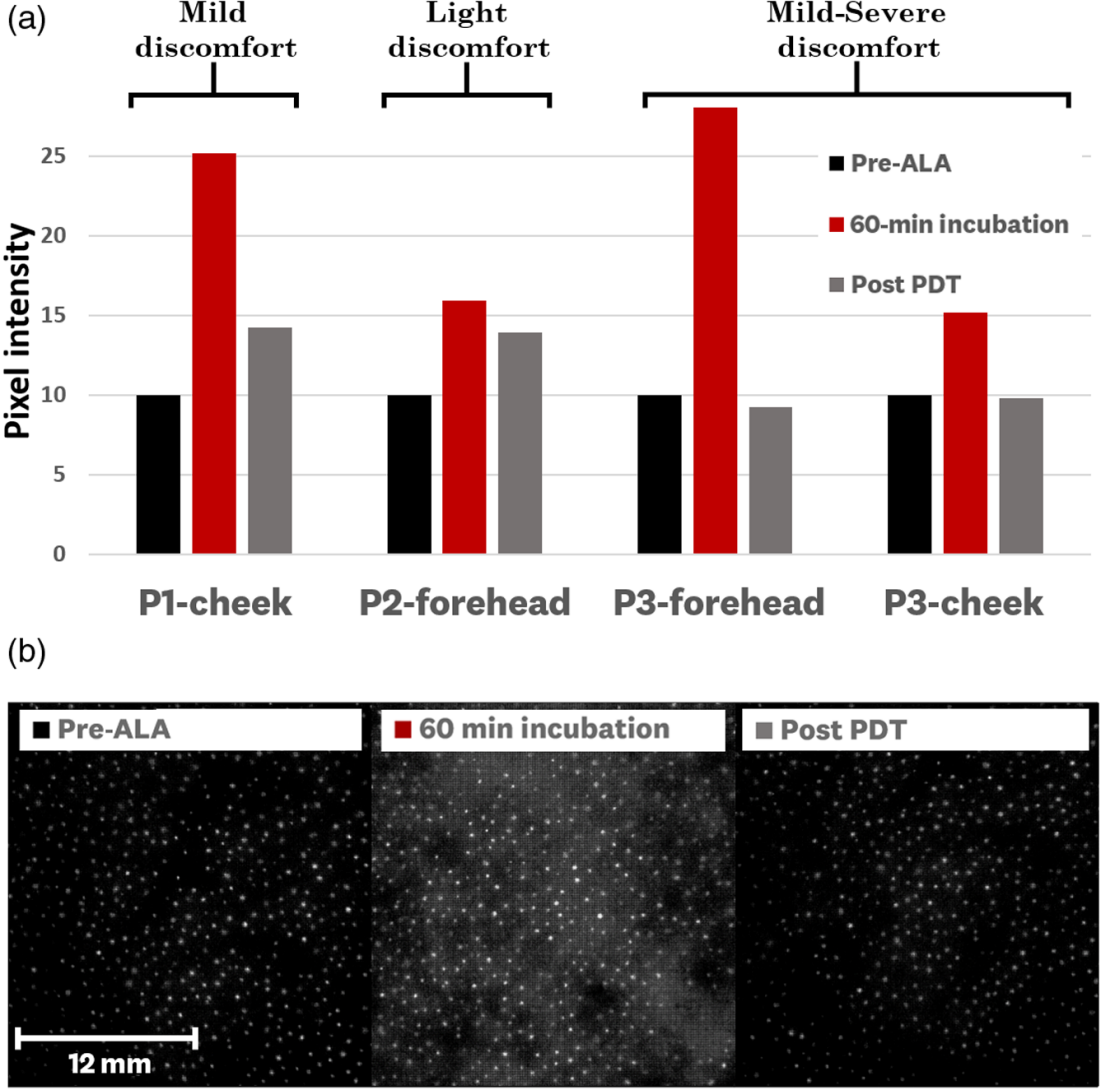
Human dosimeter measurements: results of imaging four treatment sites (three patients) during standard AK treatment at the Dartmouth-Hitchcok Dermatology Department using nonoccluded topical ALA application. Patients were imaged before ALA application, at 60-min incubation, and post-PDT treatment. (a) Average pixel values of the images are graphed for each site and baselined to the pre-ALA pixel value with a +10 value offset; the PDT-induced photobleaching measurement was positively correlated with the discomfort level reported by the patients. (b) Representative treatment image set, corresponding to patient 3 forehead, where the variation of PpIX fluorescence on the surface of the skin can be observed; it is worth noting that the bright spots on the image correspond to the sebaceous glands of the patient’s forehead.

Three patients undergoing standard AK treatment were imaged before ALA application, at 60-min incubation, and post-PDT treatment. The resulting average pixel values from each treated site ROI (four sites in total) were plotted for each image [Fig. 6(a)]. Measurements of all three patients (four sites in total) showed PpIX fluorescence accumulation as well as PDT-induced photobleaching. With the baseline (pre-ALA) value set to 10, the average 60-min incubation and post-PDT values (averaged over the four sites) were 21.1±6.1 and 11.8±2.6, respectively.

Furthermore, we observe a positive correlation between PDT-induced photobleaching and reported patient discomfort during treatment, where a higher drop in fluorescence correlated with a higher patient discomfort. Although the sample size of clinical measurements presented is small, this correlation has been reported in other publications.^18^^,^^22^^,^^35^

A representative set of images for a single site is shown in Fig. 6(b). This image set corresponds to the patient 3 forehead measurements plotted in Fig. 6(a). In this image set, we observe the heterogeneous accumulation and photobleaching of the PpIX fluorescence throughout the skin of the patient. It is worth noting that bright spots observed in the images correspond to the patient’s sebaceous glands.

## 4 Discussion

### 4.1 System Performance

The characterization tests for pixel linearity, spectral output, and excitation stability showed the system’s ability to overcome measurement reproducibility challenges faced by previously reported quantitative dosimetry systems.^32^ Concentration sensitivity tests demonstrated the dosimeter’s ability to linearly measure PpIX concentrations over 2 orders of magnitude, as shown in Fig. 4, with a sensitivity of detection down to 0.01  μM and a maximum linear detection range defined near 2  μM. These concentrations are the PpIX ranges expected in tissue, based on previous fiber probe measurements,^5^ and so it was concluded that the dosimeter’s combination of the 405-nm LED ring, measurement base, 12-bit RAW imaging format, fixed imaging distance, and double-filtered red emission, was suitable for *in-vivo* imaging.

### 4.2 *In-Vivo* Demonstrations of Performance and Use

The data presented in Figs. 5 and 6 clearly indicate that there are easily measurable signals of PpIX within minutes of application of either Ameluz^®^ (on mouse skin) or Levulan^®^ (on human skin). In the case of human studies, both PpIX accumulation and PDT photobleaching were captured for all the four skin sites measured. The ALA-produced PpIX had high variability between sites (21.1±6.1 standard deviation), and the photobleaching of it was nearly complete after treatment (falling to an average of 11.8±2.6 after PDT, where the pre-ALA baseline was 10.0). Both of these facts, variability of production and photobleaching from PDT, are well known in the published literature, but the measurements here indicate that this can be quantified and even spatially analyzed with the smartphone dosimeter.

Furthermore, these wide-field images emphasize how point-probe measurements provide insufficient spatial information to account for variations of PpIX fluorescence throughout the skin. It is worth noting that bright spots observed in the images [Fig. 6(b)] correspond to the patient’s sebaceous glands, such that future work could be focused around spatial processing of these images to separate background fluorescence from ALA-induced fluorescence.

These clinical measurement results showcase the smartphone dosimeter’s ability to capture PpIX accumulation and PDT-induced photobleaching within standard PDT treatment protocols, providing a translational tool that can be easily incorporated in clinical studies. The next phase for the smartphone dosimeter is clinical studies that assess the wide-field PpIX accumulation and PDT-induced photobleaching correlation with patient outcomes to establish treatment thresholds. With these established thresholds, treatment planning can be incorporated within the iOS application to generate a streamlined clinical workflow for individualized guided PDT treatment.

### 4.3 Dosimeter Design Improvements

Based on the characterization, preclinical, and clinical measurements, changes to the dosimeter design that would allow for improved performance include (1) fine-tuning of the emission filter long-pass wavelength to balance the trade-off between signal-to-noise and signal-to-background ratios and (2) implementation of light-source control within the iOS-app for automatic “flash” triggering during imaging. Both of these improvements will be realized in a future version of the system.

Changes to the design that would allow for “cost reduction” include using a gel filter as an alternative to the dielectric filter long-pass filter. Measurement of gel filters (i.e., 780 AS Golden Amber, LEE Filters Inc.) showed the ability to have comparable OD stopband performance to dielectric filters with the caveat of having a lower pass-band transmission and a longer transition band. Furthermore, implementation of a custom board that integrates battery management and the constant current driver could also allow for cost reduction and further miniaturization of the device.

## 5 Conclusions

In this study, the first low-cost quantitative wide-field fluorescence imaging system for PpIX-PDT skin dosimetry, designed for use in clinical studies and for easy translation into standard clinical workflow, was tested for measurement reproducibility and sensitivity as well as in preclinical and clinical environments. Characterization tests of the smartphone-based dosimeter showed its ability to overcome measurement reproducibility issues encountered by previous quantitative systems as well as the ability to image clinically relevant concentrations of PpIX in the 0.01 to 2.0  μM range. Preclinical measurements of athymic nude mice captured the *in-vivo* accumulation of PpIX with topical application of ALA, showing the system’s ability to be used in preclinical skin studies. The device was also tested in a standard clinical procedure for AK lesion treatment, where it captured wide-field PpIX accumulation and PDT-induced photobleaching. These results lay the foundation for using the system in clinical studies to establish treatment thresholds for the individualization of PDT treatment. The ultimate goal is the implementation of these thresholds alongside the system’s dosimetry to create a translational workflow that guides critical treatment parameters at the point of care for improved patient outcomes, including increased clearance rates and reduced treatment pain.
